# Successful pediatric heart transplantation with bivalirudin use in a cyanotic single ventricle patient with an intracorporeal continuous flow ventricular assist device and heparin-induced thrombocytopenia

**DOI:** 10.1007/s12055-024-01892-6

**Published:** 2025-01-07

**Authors:** Sandeep Sainathan, Leonardo Mulinari

**Affiliations:** 1 Division of Pediatric Cardiothoracic Surgery, University of Miami, Miami, USA FL; 2 Miami, USA

**Keywords:** Single ventricle congenital heart disease, Heparin-induced thrombocytopenia, Ventricular assist device, Bivalirudin, Orthotopic heart transplantation

## Abstract

We describe a case of profound coagulopathy during orthotopic heart transplantation in a cyanotic single ventricle pediatric patient with an intracorporeal continuous flow ventricular assist device performed on bivalirudin for heparin-induced thrombocytopenia. This was successfully managed with central veno-arterial extracorporeal membrane oxygenation and hemofiltration as an adjunct to treat bivalirudin-induced coagulopathy due to lack of a reversal agent for bivalirudin.

## Introduction

Cardiopulmonary bypass with the use of bivalirudin as an anticoagulant is necessary in patients with type II heparin-induced thrombocytopenia (HIT) [1–3]. Since there is no reversal agent for bivalirudin, managing coagulopathy post-cardiotomy can be challenging, especially with impaired renal function [1, 4]. We describe a case of profound coagulopathy during orthotopic heart transplant (HTX) in a patient with single ventricular physiology with a ventricular assist device (VAD) performed on bivalirudin for HIT which was successfully managed with central veno-arterial extracorporeal membrane oxygenation (VA ECMO) and hemofiltration as an adjunct to treat bivalirudin-induced coagulopathy due to lack of a reversal agent for bivalirudin.

## Case report

**Fig. 1:**
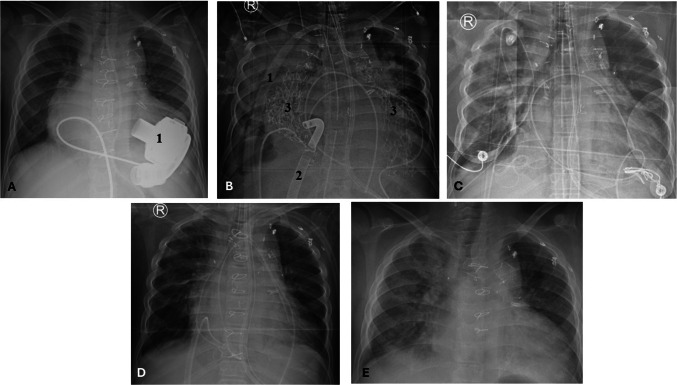
**A** Chest X-ray (CXR) showing HeartMate 3 ventricular assist device (1). **B** CXR showing Veno-arterial cannula (1—arterial, 2—venous) for extracorporeal membrane oxygenation (ECMO), QuickClot Gauze (3). **C** CXR post-ECMO decannulation and temporarily chest closure. **D** CXR post-definitive chest closure. **E** CXR 2 years post-heart transplant

**Fig. 2:**
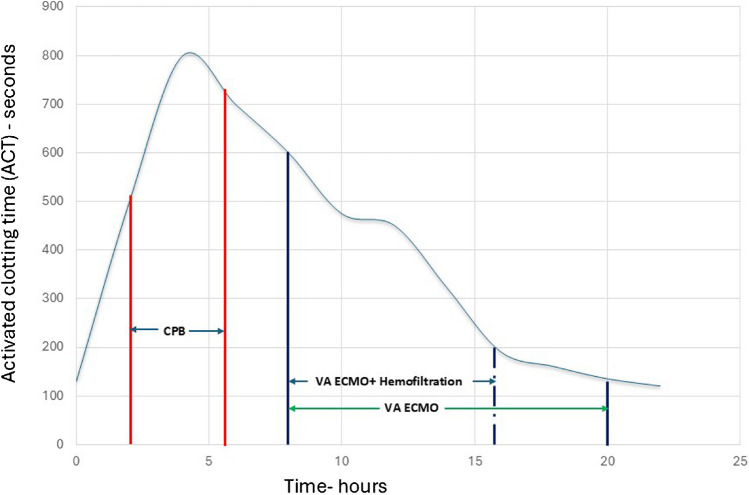
Trajectory of activated clotting time pre-, during, and post-cardiopulmonary bypass on bivalirudin

The patient is a 9-year-old, 25-kg male with hypoplastic left heart syndrome. His single ventricle palliation failed after the second stage of superior cavopulmonary anastomosis due to poor single ventricular function with a baseline arterial saturation of 75–80%. The patient underwent a HeartMate 3 VAD (Abbott, Green Oaks, IL) implant as a bridge to an HTX (Fig. 1A-1) and the implant technique has been previously described [5]. After the implant, he developed type II HIT evidenced by a positive anti-platelet factor-4 antibody, positive serotonin-release assay, and multiple venous thrombosis including partial thrombosis of the superior cavopulmonary anastomosis. He was anticoagulated with bivalirudin (Angiomax, The Medicines Company, Parsippany, NJ). Then, he transitioned to warfarin for a target International Normalized Ratio (INR) of 2.5 for the VAD and the venous thrombosis, while awaiting a HTX as a United Network for Organ Sharing (UNOS) Status 1A. The patient had chronically impaired renal function with a baseline serum creatinine of 0.7 mg/dl with episodes of acute renal failure with a peak serum creatinine of 2.4 mg/dl. After 6 months of waiting, a suitable donor heart became available. The INR was 2.2 on warfarin and was reversed with intravenous vitamin K and fresh frozen plasma. A fifth-time redo-sternotomy was performed and given HIT; bivalirudin was used for the conduct of the cardiopulmonary bypass (CPB). Bivalirudin was bolused at 1 mg/kg and maintained with a 2.5-mg/kg/h infusion for an activated clotting time (ACT) > 400 s. Precautions specific to CPB run on bivalirudin such as avoiding pooling of blood, cardiotomy suction use only while on bypass, and use of a recirculating line in the CPB circuit with cessation of bypass were observed to prevent coagulation from stagnation of blood. Due to the vascularized chest wall-based mediastinal adhesions from the chronic cyanotic state and the presence of an implanted VAD, the operative field was challenging. The branch pulmonary arteries were reconstructed with a Gore-Tex® patch (Gore, Flagstaff, AZ) after taking down the superior cavopulmonary anastomosis. The rest of the implant was a standard bi-caval technique of orthoptic HTX. The allograft ischemic time was 270 min. The heart was reperfused for 40 min commiserate with allograft ischemic time and during this bivalirudin infusion was stopped 15 min before termination of CPB to allow for a wear-off. The patient was gradually weaned off CPB with good allograft function and the CPB time was 300 min. However, the patient was diffusely coagulopathic with ACT in the 800 s. Typically, the ACT would be expected to downtrend as the bivalirudin spontaneously metabolizes with a half-life of 25–30 min, especially with preserved renal function. However, the ACT was 800 s with diffuse coagulopathic bleeding and no anastomotic site bleeding. The bleeding was profuse, needing constant transfusion of red blood cells and products and given the significant volume shifts, and modified ultrafiltration could not be performed. The coagulopathy was further exacerbated by the extensive raw surface area from the redo nature of the surgery in a patient with a VAD and divided vascularized adhesions from a chronically cyanotic state. Correction of the ACT with blood products, factor VII concentrate (NovonSeven®RT, Novo Nordisk, Plainsboro, NJ) was only partially successful in correcting it to 600 s suggesting a persisting bivalirudin effect. Given the need for continued blood product transfusion and its negative impact on the freshly implanted allograft, waiting for bivalirudin to further spontaneously metabolize would not be practical. The mediastinal space was packed tightly with QuickClot Z-fold gauze (Teleflex, Morrisville, NC) with local hemostatic properties from kaolin (Fig. 1B-3). The patient was transitioned to central VA ECMO with a right atrial outflow cannula and an ascending aorta inflow cannula, and the chest was temporarily closed with multiple mediastinal drains (Fig. 1B-1,2). Since the allograft function was good and the heart was ejecting, no left-sided venting was required. A PrismaFlex (Baxter, Deerfield, IL) renal replacement therapy circuit was spliced into the ECMO circuit, and hemofiltration was started with a net even balance. The patient was moved to the pediatric cardiac intensive care unit. The patient was kept normothermic. The serum hematocrit was kept > 30 gm% and platelet count > 100,000 cells/mm^3^. With the hemofiltration, the ACT gradually improved in the next 8 h to < 200 s. At this point, the hemofiltration was discontinued. Once the ACT reached 140 s, 20 h after surgery, the chest was reexplored at the bedside, the packs were removed, and the VA ECMO was discontinued (Fig. 1C). The ACT values during the CPB run and afterward are shown (Fig. 2). The allograft function was excellent with good hemodynamics and the mediastinum was hemostatic. The chest was left temporarily closed and definitive closure was performed 48 h later (Fig. 1D). The patient gradually convalesced and was discharged 45 days after the transplant and is thriving at 2 years post-transplantation (Fig. 1E).

## Discussion

HTX in a single ventricle cyanotic patient with a VAD can be challenging. Besides the presence of vascularized chest-wall-based adhesion from the chronic cyanotic state, adhesions induced by an intracorporeal VAD, platelet dysfunction from a chronic continuous flow VAD and renal insufficiency, and coagulation abnormalities from chronic cyanosis can predispose the patient for a post-operative hemorrhage [6, 7]. The bleeding susceptibility is further increased when the operation needs to be performed on bivalirudin as there is no reversal agent for it.

Bivalirudin is a direct thrombin inhibitor. It does not activate platelets and is used in type II HIT where there is an immune-based activation of platelets by heparin leading to arterio-venous thrombosis [4]. Thus, it is used as a systemic anticoagulant for the conduct of CPB in patients with HIT [1, 3]. Unlike heparin, there is no reversal agent for it, and it is predominantly cleared by plasma proteases (80%), with renal clearance (20%) having a smaller contribution [1]. The half-life of bivalirudin is 25–30 min. However, this can be substantially prolonged to 60 to 180 min with diminishing renal function [4]. After the conduct of CPB, bivalirudin is expected to clear in two-half lives, about 1 h after stopping the infusion with a normal renal function [1]. An alternative to bivalirudin is argatroban, another direct thrombin inhibitor. It is less preferred in HTX due to a less predictable dose response, its dependence on hepatic clearance which may be impaired due to congestion from right ventricular dysfunction after an HTX, longer half-life, and no option for hemofiltration for clearance [2].

Compared to unfractionated heparin, where its anticoagulant effect can be immediately reversed with protamine, bivalirudin has no reversal agent. When used for CPB, its effect ceases by proteolytic metabolism in the plasma and renal excretion when the infusion is stopped. Hence, it is recommended to discontinue the bivalirudin infusion about 15 min before weaning off CPB to allow for the proteolytic metabolism and renal excretion to be enhanced by modified ultrafiltration after termination of CPB to achieve hemostasis [1]. In our case, with extensive vascularized raw surface area causing signification bleeding, the possibility of deficient plasma proteases, impairment of the coagulation system from the chronic cyanosis, platelet dysfunction from chronic renal insufficiency and a continuous flow VAD, and the inability to perform modified ultrafiltration due to significant volumes changes from the bleeding, the bivalirudin effect persisted causing a serious feedback cycle of ongoing coagulopathy. If the rate of bleeding is slow, time can be given for the bivalirudin effect to wear off by enzymatic degradation and can be further enhanced by ultrafiltration. This strategy was followed after HTX in a cyanotic pediatric patient without an intracorporeal VAD and it took 12 h to achieve satisfactory hemostasis [2]. In our patient, the profuse non-surgical bleeding was controlled by tightly packing the pericardial space with a kaolin-impregnated, factor XII activating locally hemostatic gauze (QuickClot Z-fold), and the patient was transitioned to central VA ECMO with a hemofiltration device spliced in for removal of the bivalirudin. Given the coagulopathy, the ECMO was run without any anticoagulant, and with this, the bleeding rate was manageable, and the patient could be safely transported to the pediatric cardiovascular intensive care unit and kept normothermic, and satisfactory hemostasis was achieved in 8 h. The allograft and the lungs were protected from damage secondary to transfusion of large volumes of blood products and are known to be deleterious after an HTX [8]. There is always some degree of right ventricular dysfunction after an HTX which can exacerbate bleeding by central venous pressure elevation, and this was negated by VA ECMO support. In addition, there was renal protection due to stable hemodynamics with VA ECMO use.

In the published literature, five heart transplants on bivalirudin have been reported with two being pediatric transplants [3]. None of the transplants was done in a cyanotic patient with an intracorporeal chronic VAD. In most, the bleeding was manageable and was supported with blood product transfusion, and hemofiltration until the bivalirudin effect wore off.

## Conclusion

This case represents a novel application of central VA ECMO with a hemofiltration circuit as an adjunct to manage to propound coagulopathy in a pediatric patient undergoing HTX with bivalirudin.

## Data Availability

All patient anonymised data shall be available on reasonable request.
